# Use of Smokeless Tobacco Before Conception and Its Relationship With Maternal and Fetal Outcomes of Pregnancy in Thatta, Pakistan: Findings From Women First Study

**DOI:** 10.1093/ntr/ntaa215

**Published:** 2020-10-21

**Authors:** Sumera Aziz Ali, Umber Khan, Farina Abrejo, Brandi Vollmer, Sarah Saleem, K Michael Hambidge, Nancy F Krebs, Jamie E Westcott, Robert L Goldenberg, Elizabeth M McClure, Omrana Pasha

**Affiliations:** 1 Department of Epidemiology, Columbia University New York, NY, USA; 2 Department of Community Health Sciences, Aga Khan University, Karachi, Pakistan; 3 Department of Pediatrics, Section of Nutrition, University of Colorado Anschutz Medical Campus, Aurora, CO, USA; 4 Department of Obstetrics and Gynecology, Columbia University, New York, NY, USA; 5 RTI International, 3040 E. Cornwallis Road, Research Triangle Park, NC, USA; 6 Johns Hopkins Bloomberg School of Public Health, Baltimore, MD, USA

## Abstract

**Introduction:**

Smokeless tobacco (SLT) consumption during pregnancy has adverse consequences for the mother and fetus. We aimed to investigate the effects of maternal pre-pregnancy SLT consumption on maternal and fetal outcomes in the district of Thatta, Pakistan.

**Aims and Methods:**

We conducted a secondary data analysis of an individual randomized controlled trial of preconception maternal nutrition. Study participants were women of reproductive age (WRA) residing in the district of Thatta, Pakistan. Participants were asked questions regarding the usage of commonly consumed SLT known as gutka (exposure variable). Study outcomes included maternal anemia, miscarriage, preterm births, stillbirths, and low birth weight. We performed a cox-regression analysis by controlling for confounders such as maternal age, education, parity, working status, body mass index, and geographic clusters.

**Results:**

The study revealed that 71.5% of the women reported using gutka, with a higher proportion residing in rural areas as compared with urban areas in the district of Thatta, Pakistan. In the multivariable analysis, we did not find a statistically significant association between gutka usage and anemia [(relative risk, RR: 1.04, 95% confidence interval, CI (0.92 to 1.16)], miscarriage [(RR: 1.08, 95% CI (0.75 to 1.54)], preterm birth [(RR: 1.37, 95% CI (0.64 to 2.93)], stillbirth [(RR: 1.02, 95% CI (0.39 to 2.61)], and low birth weight [(RR: 0.96, 95% CI (0.72 to 1.28)].

**Conclusions:**

The study did not find an association between gutka usage before pregnancy and adverse maternal and fetal outcomes. In the future, robust epidemiological studies are required to detect true differences with a dose–response relationship between gutka usage both before and during pregnancy and adverse fetomaternal outcomes.

**Implications:**

While most epidemiological studies conducted in Pakistan have focused on smoking and its adverse outcomes among males, none of the studies have measured the burden of SLT among WRA and its associated adverse outcomes. In addition, previously conducted studies have primarily assessed the effect of SLT usage during pregnancy rather than before pregnancy on adverse fetal and maternal outcomes. The current study is unique because it provides an insight into the usage of SLT among WRA before pregnancy and investigates the association between pre-pregnancy SLT usage and its adverse fetomaternal outcomes in rural Pakistan.

## Introduction

More than 300 million people consume smokeless tobacco (SLT) worldwide.^1,2^ SLT products are relatively cheaper than manufactured cigarettes and some are viewed as safer substitutes for smoking.^3–5^ In some areas, the use of SLT products is considered a socially acceptable cultural norm resulting in higher usage of SLT.^6–9^ There is a long history of using SLT products globally, including the United States of America, Europe, Asia, and parts of Africa with differences in the epidemiology of SLT usage in these regions.^10,11^ Consumption of SLT products is increasing, not only among men but also among women in South Asian countries.^1,12^ For example, an updated analysis of data from 127 countries reveal that more than 85% of the ST-related burden is found in South and Southeast Asia.^13^ Further, India accounts for 70% of the DALYs lost due to SLT followed by Pakistan (7%) and Bangladesh (5%).^13^ One of the main reasons for the extensive use of SLT in Pakistan is its ease of access and low prices.^14^ In Pakistan, 15% of male and 10% of females use SLT in different forms such as gutka, Nass/Naswar, paan, and betel nut.^15,16^

Although studies have evaluated the carcinogenic effects of SLT,^17,18^ there is a dearth of evidence on assessing the effect of SLT use during pregnancy, in general, and particularly before pregnancy on fetomaternal outcomes.^19,20^ The literature reveals SLT use during pregnancy may increase the risk of adverse pregnancy outcomes such as maternal anemia, miscarriage, and stillbirths, low birth weight (LBW), and decreased birth length.^21–26^ For example, a study in Sweden demonstrates that different SLT products have modest effects on fetal growth and increased risk of preterm delivery.^27^ Likewise, a study conducted in India found that SLT use is associated with an average reduction of 105 g in birth weight and a reduction in gestational age of 6.2 days.^25^A study conducted by Gupta et al. found an increased risk of LBW, preterm birth (PTB), and stillbirth among tobacco chewers when compared with nonchewers.^23^ Similarly, Pratinidhi et al. conducted a cohort study to assess the effect of tobacco use on pregnancy outcomes in Indian women.^20^ The authors found a significantly increased risk of LBW and stillbirth among tobacco users when compared with nonusers.^20^ Likewise, one systematic review and meta-analysis suggested a positive and statistically significant association between SLT use and adverse pregnancy outcomes including LBW, PTB, and stillbirths in Indian women.^28^ However, studies included in the meta-analysis had some limitations. First, there were only 3 studies that were primary conducted in India thus ignoring the effects of SLT in other countries of Asia. Second, some studies did not address potential confounders, such as age, parity, education, body mass index (BMI), and working status of the women. Besides, outcomes in these studies were measured using medical records rather than measuring outcomes directly. Moreover, these studies mainly assessed the use of tobacco during pregnancy instead of assessing the use of tobacco before pregnancy.^28^ Although these study findings support the association between SLT usage during pregnancy and adverse fetal and maternal outcomes, there is a lack of evidence regarding the association of SLT use before pregnancy (before conception) and fetomaternal outcomes. Since the exposures to SLT tend to accumulate over time before conception, the preconception period is important to capture these harmful exposures to assess their true effects on adverse outcomes. This is because women might tend to change their SLT use after they become pregnant thus preventing one from capturing actual exposure. Besides, exposures to such harmful exposures require a sufficient latent or induction period to produce the adverse effects; therefore, we aimed to measure the exposure to SLT before pregnancy instead of during pregnancy. Hence, this study aimed to investigate the association between gutka (SLT) use prior to pregnancy and maternal and fetal outcomes in Pakistan.

## Material and Methods

### Study Design and Sample Size

The sample for this secondary analysis comes from the Women First (WF) Preconception study that was conducted in the Democratic Republic of Congo, Guatemala, India, and Pakistan.^29^ This was an individually randomized, controlled efficacy trial (NCT01883193) that included three arms: women in arm 1 started a nutritional supplement ≥3 months before conception and continued through delivery; women in arm 2 started the same nutritional supplement at the end of the first trimester and continued through delivery, and women in arm 3 (control arm) received no nutrition supplements.^29^ The main objective of the WF study was “to determine the benefits to the offspring of women of commencing a daily comprehensive maternal nutrition supplement ≥3 months before conception versus the benefits of commencing the same supplement at 12–14 weeks gestation and also to compare offspring outcomes with those of a third control arm.”  ^29^ Sample size of the primary WF study was based on having 80% power and maintaining a study-wide type I error of 0.05 across all planned primary hypothesis tests.^29^ Assuming an α-level of 0.00625, a two-sided test, and an SD of 1.0 for the primary outcome (birth length), 192 women per arm in each country were needed to detect an effect size of 0.37 with 80% power for the primary outcome of birth length in the main study. Around 240 women were required per arm enter phase 2 (pregnancy) within each country after accounting for 20% attrition during pregnancy. Furthermore, this number was increased to 480 women per arm to be enrolled in each country, assuming that only 50% of women enrolled would become pregnant.^29^ Further details of the WF study are discussed in depth elsewhere.^29,30^

Since the WF study was not designed to assess the effect of gutka usage on adverse fetomaternal outcomes, we did a power analysis to evaluate whether the sample size for the current study was sufficient to answer the research question. We did power analysis for adverse outcomes reported commonly in the literature such as LBW, PTB, and stillbirth using the effect size from a recently conducted systematic review and meta-analysis.^28^ The findings of the power analysis revealed that our study was powered appropriately to detect the desirable effect sizes of 1.88 for LBW, 2.54 for PTB, and 2.85 for stillbirths.^28^ More specifically, considering the proportion of various outcomes in our study population (LBW: 15% to 20%; PTB: 15% to 17%; stillbirth: 3.4% to 5.6%; pregnancy loss or miscarriage: 15% to 16%; anemia: 50% to 75%),^31–33^ having 80% power and maintaining a level of significance as 5% we need to have a minimum of 65 to 175 women per group. Our study had at least 197 women in each group, thus was sufficiently powered to detect the true difference between gutka users and adverse fetal and maternal outcomes.

### Study Participants

Study participants were married WRA residing in the district of Thatta, Pakistan. Women were identified through tracking women in a maternal and newborn health registry that is active in Thatta district, in addition to household surveys, health care facilities, word-of-mouth, and meetings with the traditional birth attendants and community leaders.^29,30^ Women were eligible to participate in the primary WF trial if they were 16–35 years old; had 0–5 children, were not using or planned to use family planning methods; and planned to conceive during the following 18 months.^29,30^

### The Flow of Study Participants During Different Phases of the Study

**Figure 1.**
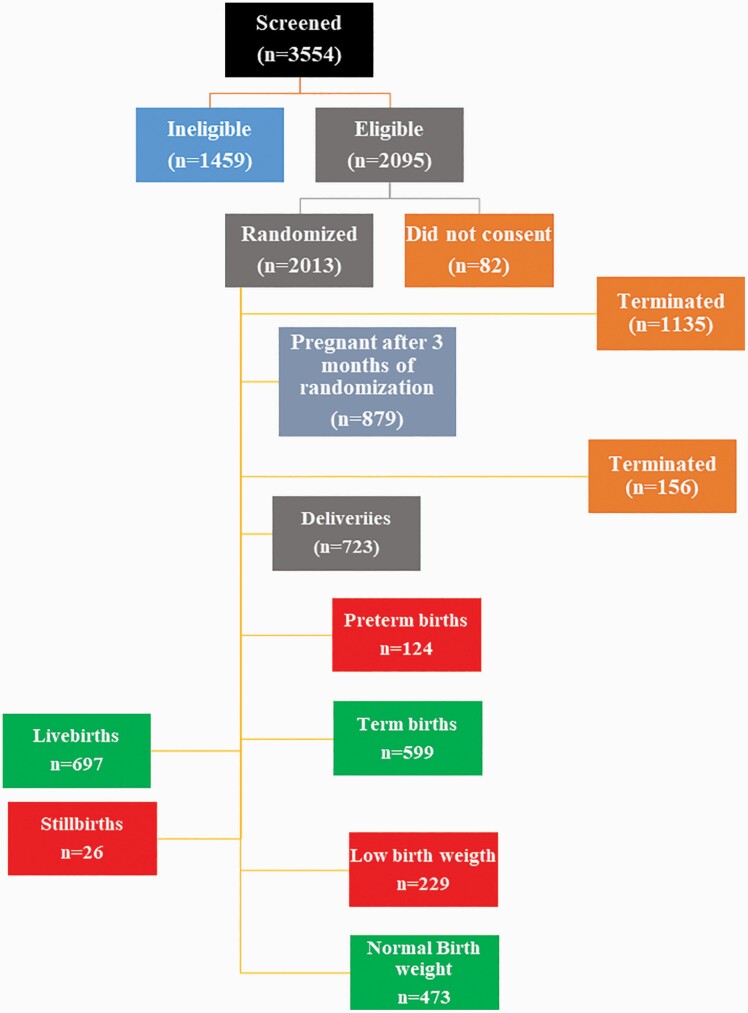
Flow chart of woman first study participants in district Thatta, Pakistan.

Figure 1 depicts the flow of participants that were included in this secondary data analysis. At the enrollment, we screened 3554 married and nonpregnant women. Of those, 2095 women were eligible for the study.^29^ Of the eligible women, 2013 women agreed to participate and were enrolled. Data on gutka consumption were collected from 2013 women before they became pregnant. These 2013 women were followed longitudinally unless they became pregnant within 3 months of enrolment. This number decreased as these women were followed until they became pregnant and delivered their babies (Figure 1). As per the protocol of the main WF study, women had to become pregnant after 3 months of enrollment, otherwise, their participation in the study was discontinued without collecting any further data from them. Of 2013 women, the participation of 1135 women was discontinued, with the most common reason being pregnancy within 3 months of enrolment (90%). Thus, out of 2013 women, 879 (43.6%) women became pregnant after 3 months of enrollment, and they were followed until the end of their pregnancy. After pregnancy, maternal and fetal outcomes of 879 pregnant women were monitored and recorded during biweekly home visits. After becoming pregnant, the participation of 156 additional women (17.7%) was discontinued, mostly due to early pregnancy loss/miscarriage before 20 weeks of gestation (*n* = 152). Thus, 723 (82.2%) women were followed until they delivered their baby. Of these, 124 (17.1%) women delivered before and 599 women (82.8%) delivered their babies after 37 weeks of gestation (full-term deliveries). Of the 723 deliveries, 26 (3.4%) were reported as stillbirths. Birth weight data were available for 702 babies (97%) and 229 (31.6%) babies were found to have a LBW (<2500 g) and 473 (67.3%) had a normal weight of more than 2500 g. Due to the longitudinal nature of the study and the range of fetomaternal outcomes, the sample size varied for different outcomes in the analysis. For example, the data were collected for maternal anemia at the enrollment and, therefore, data were available for 2013 women to assess the association between gutka and maternal anemia. On the other hand, data for pregnancy loss were only available for 879 women, as these were the women who became pregnant after 3 months of enrolment and were followed until delivery or pregnancy loss. Due to pregnancy losses, not all women were followed until delivery; therefore, data for the outcome of stillbirth and preterm delivery were available for 723 women. Finally, we were able to collect data on birth weight for 700 live births and two stillbirths; therefore, the sample size for assessing the relationship between gutka usage and LBW was 702.

### Study Setting and Data Collection

Although it was a multi-country study, we only analyzed data from Pakistan for the current study. In Pakistan, we implemented this study across 14 geographic areas of district Thatta including Gujjo-1 (ID:921), Mirpur Sakro (ID: 926), Ghulamullah 1 (ID: 915), Karampur (ID: 911), Kalari (ID:911), Gujjo-2 (ID:932), Ghullamullah-2 (ID:931), Chatochand (ID:918), Mirpur Sakro-2 (ID:934), Makli cluster (ID:912), Thatta-1 (ID:913), Thatta-2 (ID:919), Sujawal (ID:924), and Gharo (ID:916).^34^ We included all women who completed the WF primary study and had delivery outcomes recorded. At the time of enrollment, we asked questions from women about their socio-demographic characteristics, food insecurity, indoor air pollution, obstetric history, and maternal health status. We measured their weight, height, BMI (in kg/m^2^), waist circumference, and hip circumference before they became pregnant. Field workers administered a urine pregnancy test to confirm the pregnancy status of the women if a woman had missed their periods, which was followed by conducting an ultrasound to confirm the pregnancy. We assessed SLT usage by asking the question “Do you currently chew tobacco” (Yes or No). Additionally, women may have continued consuming tobacco during pregnancy, or some may have stopped consuming tobacco due to their pregnancy. Since we did not collect the data of tobacco use during the pregnancy, this study assessed only the association between pre-pregnancy gutka usage and adverse fetomaternal outcomes.

### Primary Exposure and Outcome Variables

The main exposure variable for this analysis was the use of gutka (the most common type of SLT) before pregnancy. The main fetomaternal outcomes assessed included maternal anemia: hemoglobin (Hb levels of <12 g/dL^35^; miscarriage: unintended loss of a fetus before the first 20 weeks of gestation as determined by the reported last menstrual period and confirmed by ultrasound; PTB: delivery at <37 weeks of gestational age confirmed by ultrasound^36^; stillbirths defined as the birth of a dead fetus at the gestation week of 20 or more confirmed by ultrasound; and LBW: birth weight <2500 g.^37,38^ We measured Hb levels (g/dL) of women by using HemoCue 201machines (HemoCue, Brea, CA). At the time of analysis, we categorized Hb into a binary variable (anemic: <12 g/dL and nonanemic: ≥12 g/dL).^35^

### Confounders

Based on the literature review potential confounders included were mother’s age (years), parity (number of children ever born after 20 weeks of gestational age), which was categorized into nulliparous (no child born), one to four children, or at least five children, mother’s education (illiterate or literate), current working status (Yes/No), cooking place (separate building to cook, cooking takes place outdoors, or in the house), food insecurity (Yes/No), place of residence (urban/rural). BMI was calculated as a continuous variable by taking a ratio of weight (in kilograms) to the square of height (in meters). We categorized BMI as underweight (<18.5 kg/m^2^), normal (18.5–24.9 kg/m^2^), overweight or obese (≥25.0 kg/m^2^).

### Data Management

Two mechanisms were placed for data management, one in the field in Thatta and the other in the Aga Khan University (AKU), Karachi, Pakistan located at the distance of 98 km from Thatta. We hired five experienced field supervisors, who supervised field workers. These field supervisors checked the filled data collection forms randomly and edited the forms every week before transmitting to AKU for second editing. In the field, the forms were edited, and an error list was generated by the field supervisors to be corrected in the field by re-visiting the participant to address the missing information and inconsistent errors. Besides, the principal investigator (PI) made weekly random visits to assess the quality of data being collected in the field. The completed data collection tools were returned to the data management system at AKU, Karachi, Pakistan. A data quality assurance officer evaluated forms a second time to ensure that all data were collected from the participant before she handed over forms to the data entry operators who entered forms in standardized data entry templates. The data manager and PI reviewed the entered data and assisted in data cleaning before sending the data to the Research Triangle Institute, Intl (RTI) located in North Carolina, USA for further quality checks. A monthly monitoring report was generated and shared by RTI with the PI for making necessary corrections in the data before analysis. These monthly RTI reports helped PI to plan for refresher training whenever necessary to improve data quality.

### Statistical Analyses

Frequencies with proportions were calculated for users and nonusers of gutka. A Chi-square test was used to compare gutka users and nonusers, for sociodemographic and nutritional characteristics of the study participants. Statistical significance was set at the 5% level. To assess the association between gutka usage before the pregnancy and fetomaternal outcomes, we used Cox-regression analysis to calculate the relative risks (RR) with their 95% confidence intervals (CIs).

We constructed five series of multivariable models to assess the effect of gutka on five outcomes including anemia, pregnancy loss, PTB, stillbirth, and LBW separately. Model series 1 assessed the association between gutka usage and maternal anemia after controlling for mother’s age, parity, mother’s education, working status, cooking place, BMI, and geographic areas. Model series 2 and 3 assessed the association between gutka usage and pregnancy loss and PTB, respectively. These two models controlled for all the same confounders as model series 1, but additionally adjusted for maternal anemia. Model series 4 assessed the association between gutka and stillbirth while controlling for all the variables included in models 1, 2, and 3 and additionally adjusted for gestational age. Finally, model series 5 assessed the association between gutka usage and LBW after controlling for mother’s age, parity, mother’s education, working status, cooking place, BMI, and geographic areas. Statistical analyses were conducted using SPSS Version 20 (IBM SPSS Statistics for Windows 20.0, Armonk, NY).

### Ethical Approval

The ethics review committees of Aga Khan University and the institutional review board of the University of Colorado Denver approved the study. All women provided written informed consent.

## Results

### Sociodemographic and Nutritional Characteristics of Study Participants

Table 1 shows more than 50% of the women were <25 years old and more than 80% of the women were illiterate. Regarding working status, 14% of the women and more than 90% of their husbands reported working for earning, respectively. More than a third of the women (38%) were nulliparous and 56% of the women had one to four children. Nearly half of the women (48%) reported cooking in a separate building and 44% reported cooking outdoor. For nutritional characteristics, nearly three-fourths of the women (73%) were found to be anemic, about one-third of the women (35%) had a BMI of <18.5 kg/m^2^, and around half of the women (45.4%) reported food insecurity in their houses.

**Table 1.** Sociodemographic and nutritional characteristics for women of reproductive age at the time of enrolment in the Women First Study in Thatta Pakistan (*n* = 2013)

Characteristics	To tal	Exposed	Nonexposed
	*n* (%)	(Gutka usage)*n* (%)	(No. of gutka usage)*n* (%)
Age (years)			
<25	1109 (55)	795 (55)	314 (54.7)
≥25	904 (45)	645 (45)	259 (45.3)
Educational status of woman			
Literate	366 (18)	272 (18.9)	94 (16.4)
Illiterate	1647 (82)	1168 (81.1)	479 (83.6)
Working status of women			
Yes	280 (14)	207 (14.4)	73 (12.7)
No	1733 (86)	1233 (85.6)	500 (87.3)
Husband’s work status			
Yes	1863 (92)	1332 (92.5)	531 (92.7)
No	150 (8)	108 (7.5)	42 (7.3)
Food insecurity			
Yes	913 (45.4)	665 (46.2)	248 (43.3)
No	1100 (54.6)	775 (53.8)	325 (56.7)
Parity			
Nulliparous	761 (38)	562 (34.7)	199 (34.7)
1–4	1118 (56)	788 (54.7)	330 (57.6)
≥ 5	134 (6)	90 (6.2)	44 (7.7)
Cooking place			
Separate building	973 (48)	714 (49.6)	259 (45.2)
Outdoor	881 (44)	613 (42.6)	268 (46.8)
In the house	159 (8)	113 (7.8)	46 (8.0)
BMI (kg/m^2^)			
<18.5	707 (35)	515 (34.7)	192 (36.2)
18.5–24.9	1154 (58)	853 (57.5)	301 (56.8)
≥ 25	152 (7)	115 (7.8)	37 (7)
Maternal anemia (Hb: g/dL)			
Yes (<12 g/dL)	1483 (73)	1069 (74.2)	414 (72.3)
No (≥12 g /dL)	530 (27)	371 (25.8)	159 (27.7)

*p*-value for Chi-squared test was >0.05 for all these variables in Table 1

**Figure 2.**
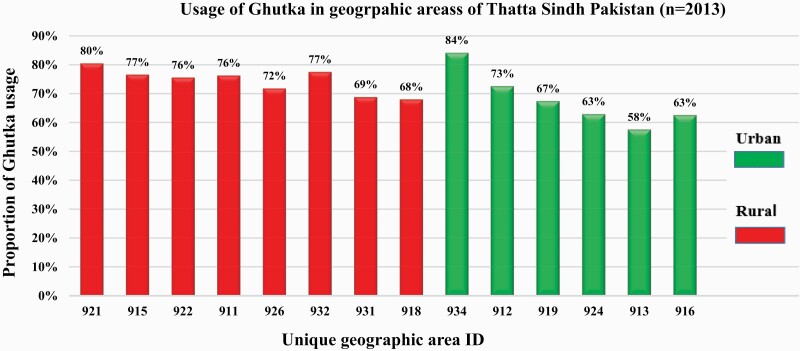
Use of gutka in different geographic areas (urban and rural) at the time of enrollment in the woman first study in district Thatta, Pakistan (*n* = 2013).

The data suggest that 1440 women (71.5%) reported using gutka in district Thatta, while 573 (28.5%) women were nonusers. Gutka users resided more in the rural areas, ranging from 68% to 80% with an average of 74% when compared with the urban areas, where the proportion of gutka users varied from 63% to 84% with an average of 68% (Figure 2). We did not find any differences between users/nonusers of gutka for sociodemographic variables among WRA (Table 1).

### Fetomaternal Outcomes Associated with Gutka Usage and Other Important Factors

Around three-fourths (74.2%) of the gutka users and 72.3 % of nonusers were anemic (*p*-value: > 0.05). There was no difference between gutka users and nonusers for any maternal and fetal outcomes as shown in Table 2.

**Table 2.** Adverse fetal and maternal outcomes associated with gutka usage (main exposure) among married women of reproductive age enrolled in WF study in district—Thatta Pakistan

Fetal and maternal outcomes	Use of gutka		Crude RR, 95% CI^	Adjusted RR, 95% CI^^
	Exposed	Unexposed		
Anemia (Hb: g/dL)				
No (<12 g/dL)	371 (25.8)	159 (27.7)	1	1
Yes (≥12 g/dL)	1069 (74.2)	414 (72.3)	1.04 (0.93–1.17)	‡1.02 (0.91–1.14)
Pregnancy loss				
No miscarriage	529 (82.7)	199 (82.9)	1	1
Miscarriage	111 (17.3)	41 (17.1)	1.01 (0.69–1.43)	**1.08 (0.75–1.54)
Gestational age at the time of delivery				
Term (≥37 weeks)	431 (82.4)	168 (84.0)	1	1
Preterm (<37 weeks)	92 (17.6)	32 (16.0)	1.12 (0.74–1.67)	**1.37 (0.64–2.93)
Birth				
Live birth	505 (96.2)	195 (97.0)	1	1
Stillbirth	20 (3.8)	6 (3.0)	1.31 (0.52–3.25)	*1.02 (0.39–2.61)
Birth weight				
Normal weight (≥2500 g)	344 (68.1)	130 (66.0)	1	1
Low birth weight (<2500 g)	161 (31.9)	67 (34.0)	1.09 (0.80–1.48)	‡1.11 (0.80–1.53)

^Univariable analysis.

^^Multivariable analysis.

^‡^Adjusted for mother’s age, parity, mother’s education, working status, cooking place, BMI, food insecurity, and place of residence.

**Adjusted for the above variables (^‡^) and anemia status at the baseline.

*Adjusted for the above variables (^‡^) and gestational age at the time of delivery.

The univariable analysis showed that gutka usage was not significantly associated with any of the adverse maternal and fetal outcomes (Table 2). Likewise, in the multivariable analysis, after adjusting for maternal age, education, parity, working status, BMI, geographic area, and treatment arm, we did not find any significant association between gutka usage with maternal and fetal outcomes such as anemia [(RR: 1.04, 95% CI (0.92 to 1.16)], miscarriage [(RR: 1.08, 95% CI (0.75 to 1.54)], PTB [(RR: 1.37, 95% CI (0.64 to 2.93)], stillbirth [(RR: 1.02, 95% CI (0.39 to 2.61)], and LBW [(RR: 0.96, 95% CI (0.72 to 1.28)]. The multivariable analysis also did not find a significant association of other sociodemographic and nutritional factors with different fetomaternal outcomes as shown in Table 3. However, we found a significant and positive association of food insecurity with anemia [(RR: 1.23, 95% CI (1.15 to 1.28)].

**Table 3.** Multivariable adjusted estimates (RR)* for adverse fetomaternal outcomes by various socio-demographic and nutritional characteristics among women of reproductive age enrolled in the WF study in district—Thatta Pakistan

	Maternal anemia*n* = 2013			Miscarriage*n* = 880			Preterm birth*n* = 723			Stillbirth*n* = 726			LBW*n* = 702		
Characteristics	RR	95% CI		RR	95% CI		RR	95% CI		RR	95% CI		RR	95% CI	
Women’s age (years)															
<25	1			1			1			1			1		
≥25	1.03	0.93	1.14	1.11	0.81	1.53	1.01	0.68	1.47	1.19	0.53	2.68	0.88	0.66	1.18
Parity															
Nulliparous	1			1			1			1			1		
1-four	0.98	0.88	1.09	0.96	0.67	1.35	1.07	0.72	1.58	0.71	0.31	1.63	1.03	0.76	1.38
≥5	1.01	0.82	1.25	0.56	0.25	1.23	0.83	0.35	1.96	0.42	0.05	3.31	0.63	0.3	1.31
Women’s education															
Literate	1			1			1			1			1		
Illiterate	0.94	0.83	1.08	0.86	0.58	1.28	0.96	0.59	1.57	1.01	0.33	3.05	0.77	0.55	1.07
Women’s working status															
No	1			1			1			1			1		
Yes	1.02	0.88	1.18	1.25	0.82	1.91	0.97	0.56	1.68	1.07	0.36	3.15	1.02	0.68	1.52
Gutka usage															
No	1			1			1			1			1		
Yes	1.02	0.9	1.14	1.08	0.75	1.54	1.37	0.64	2.93	1.02	0.39	2.61	1.1	0.8	1.53
Food insecurity															
No	1			1			1			1			1		
Yes	1.23	1.15	1.28	1.11	0.8	1.54	0.98	0.66	1.45	0.96	0.41	2.22	0.94	0.7	1.26
Place of residence															
Urban	1			1			1			1			1		
Rural	0.91	0.8	1.03	0.97	0.65	1.45	1.28	0.78	2.09	3.39	0.78	14.7	1.08	0.76	1.55
Cooking place															
Separate building	1			1			1			1			1		
Outdoor	0.95	0.85	1.05	0.94	0.67	1.32	0.91	0.61	1.37	1.15	0.49	2.7	1.13	0.84	1.53
In the house	0.98	0.81	1.19	0.98	0.52	1.85	1.69	0.91	3.14	0.57	0.07	4.44	1.33	0.78	2.29
BMI (kg/m^2^)															
≥ 25	1			1			1			1			1		
18.5–24.9	1.01	0.83	1.23	0.88	0.51	1.52	0.96	0.45	1.95	0.61	0.17	2.25	0.95	0.58	1.58
<18.5	0.99	0.81	1.33	0.82	0.46	1.45	0.81	0.38	1.72	0.56	0.15	2.38	0.73	0.43	1.27
Maternal anemia															
Yes (<12 g/dL)	NA			1			1			1			1		
No (≥12 g/dL)				1.03	0.71	1.49	1.06	0.67	1.66	1.07	0.36	3.15	1.07	0.76	1.49

*RR and 95% CI for each outcome was calculated using Cox-regression adjusting for main exposure of gutka and other important factors.

## Discussion

The current study examined the association of gutka usage during the pre-pregnancy period with adverse maternal and fetal outcomes in district Thatta, Pakistan. The present study depicts that around three-fourths of women consume gutka in district Thatta, Pakistan and most of them live in rural areas. Gutka users were slightly more anemic and had more stillbirths and preterm deliveries, however, results were not statistically significant. After adjusting for potential confounders, we did not find a statistically significant association between gutka usage during the pre-pregnancy period and various adverse fetal and maternal outcomes.

Our findings are consistent with some studies where maternal SLT use appears to have modest to no effect on birth weight as compared with nonusers.^39,40^ However, our study findings contradict studies where significant birth weight deficits are found among SLT users during pregnancy.^25,27,41^ For instance, a population-based prospective cohort study conducted in Mumbai, India on 1217 women found an average reduction of 105 g in birth weight among users of SLT when compared with nonusers.^25^ Likewise, in Sweden, a study conducted on 12 284 women found that adjusted mean birth weight was reduced significantly in snuff users by 39 g when compared with nonusers.^27^ Furthermore, these studies found a positive and significant association between SLT and preterm and stillbirth, which differs from our study findings.^25,41^ The study conducted in Sweden found that the risk of preterm delivery was high among snuff users when compared with nonusers.^27^ Similarly, another study conducted in India on 918 women with hemoglobin data showed a positive association of SLT with anemia among pregnant women, further contradicting our study findings.^42^ Likewise, findings from a meta-analysis using three studies found a positive and significant association between SLT during pregnancy and adverse outcomes such as stillbirth, PTB, and LBW.^28^

These differences across various studies could be due to several reasons that make it challenging to study the effects of SLT on pregnancy outcomes. For example, the differences could be due to variation in the products for nicotine content, bioavailability, nicotine delivery, the presence of additives, toxin levels, and/or portion size of gutka. Furthermore, product type and timing of exposure can vary between women, making it challenging to identify cohorts of women with identical type, intensity, and duration of SLT exposure before and during pregnancy. Finally, unlike our study, most of the studies described earlier have assessed the effect of SLT usage during pregnancy on the outcomes instead of assessing the effect of SLT use before pregnancy.

We also did not find any association of other important factors with the adverse fetal and maternal outcomes except for food insecurity associated with maternal anemia. This association implies that women who faced the problem of food insecurity were 1.23 likely to develop anemia when compared with their counterparts. This finding is comparable with other studies conducted in developing countries of Asia and Africa.^43–45^ Collectively, these findings can be explained by the fact that women with food insecurity might consume inadequate diets, with a limited variety of food groups, with poor iron content and coping mechanisms during a food shortage. Besides, it is a cultural norm in Pakistan that women usually feed their husbands, children, and other family members first and consume leftover foods themselves thus resulting in anemia. Moreover, it might be possible that these women with food insecurity have low purchasing power with greater family size thus not able to purchase the nutritious food required to prevent them from becoming anemic.^46^

### Strengths and Limitations

This study has several strengths. It is the first to examine maternal and fetal outcomes among rural Pakistani women using SLT. It was conducted in an area with a high prevalence of SLT use and addresses local concerns about exposure during pregnancy to SLT. This was a population-based study with a large sample size, which measured different maternal and fetal outcomes objectively by using ultrasound (gestational age) and Hemocue machines (anemia).

This study has some limitations. First, we did not measure tobacco use during pregnancy by asking specific questions related to gutka consumption. Women might have stopped consuming gutka once they became pregnant, which is less likely because, during biweekly visits, we found women continued using gutka during their pregnancy. Since we did not collect data by asking questions about tobacco use during pregnancy, therefore, our analysis was limited to pre-pregnancy gutka usage. Second, we also did not measure any biomarkers, such as the amount of nicotine in the urine of gutka users. However, our method of assessing exposure is consistent with other longitudinal studies that have measured exposure subjectively by relying on self-reported data. Third, we did not collect information about frequency and duration as well as the composition of gutka, such as nicotine quantity in gutka. Fourth, stigmatization around tobacco use may discourage from disclosing their tobacco use due to social desirability bias, resulting in exposure misclassification. However, this was less likely in our study due to visible teeth staining due to gutka. Fifth, data on exposure was from women at baseline (*n* > 2000) but our data on fetomaternal outcomes were only on <50% of those women. Moreover, almost three-fourths of the women in our study reported using gutka; therefore, it might be difficult to delineate the effects of gutka if the majority of the women consumed gutka. Sixth, the rates of adverse maternal and fetal outcomes are high (anemia: 75%, LBW: 17.9%, PTB: 15%–17%, stillbirth: 56.5/1000 births)) in Thatta.^31–33^ Considering the multifactorial nature of the outcome, we adjusted the results of main exposure for important factors but there might be some other unknown factors to explain the likelihood of the different outcomes. To the extent that gutka tends to suppress appetite, it may also have an indirect effect of limiting dietary intake that could have impacted the outcomes with high rates of maternal undernutrition. Pakistani participants had among the lowest mean intakes of calories, protein, and key micronutrients.^47^ This is further supported by the positive impact of the nutritional intervention on birth anthropometry in this population.^30^

Despite the null findings, it is important to consider that all tobacco products contain nicotine.^48^ Data on the adverse effects of nicotine on the developing fetus are sufficient to classify nicotine as a developmental toxin.^49^ Use of any products containing nicotine likely will have adverse effects on fetal neurological development.^50^Therefore, our study findings should be interpreted with caution.

## Conclusions

Our study did not document an association between pre-pregnancy gutka usage and adverse fetal and maternal outcomes in Thatta, Pakistan. Since we did not measure the frequency and duration of gutka usage and did not assess the intake of gutka during pregnancy, findings must be interpreted cautiously. Longitudinal and robust epidemiological studies are required to detect true differences with the dose–response relationship between gutka usage before pregnancy and adverse fetomaternal outcomes. Continuing evaluation of potential associations between SLT exposure and reproductive health outcomes is necessary to develop accurate estimates of the burden of disease and public health recommendations related to gutka use.

## Supplementary Material

ntaa215_suppl_Supplementary_Taxonomy_Form

## References

[1] Suliankatchi RA, Sinha DN, Rath R, et al. Smokeless tobacco use is “replacing” the smoking epidemic in the South-East Asia region. Nicotine Tob Res. 2019;21(1):95–100. 29281083 10.1093/ntr/ntx272

[2] McHenry G, Parker SJ, Khoury D, Conway KP. Epidemiology of smokeless tobacco use in the United States and other countries. Smokeless Tobacco Products: Elsevier; 2020:39–71. doi:10.1016/B978-0-12-818158-4.00003-0.

[3] Harrell PT, Naqvi SMH, Plunk AD, Ji M, Martins SS. Patterns of youth tobacco and polytobacco usage: the shift to alternative tobacco products. Am J Drug Alcohol Abuse. 2017;43(6):694–702. 27668320 10.1080/00952990.2016.1225072 PMC5440212

[4] Renner CC, Patten CA, Enoch C, et al. Focus groups of Y-K Delta Alaska Natives: attitudes toward tobacco use and tobacco dependence interventions. Prev Med. 2004;38(4):421–431. 15020175 10.1016/j.ypmed.2003.11.005

[5] Schensul JJ, Nair S, Bilgi S, et al. Availability, accessibility and promotion of smokeless tobacco in a low-income area of Mumbai. Tob Control. 2013;22(5):324–330. 22387521 10.1136/tobaccocontrol-2011-050148 PMC4644352

[6] Huque R, Zaman MM, Huq SM, Sinha DN. Smokeless tobacco and public health in Bangladesh. Indian J Public Health. 2017;61(suppl 1):S18–S24. 28928314 10.4103/ijph.IJPH_233_17 PMC6349136

[7] Shah S, Dave B, Shah R, Mehta TR, Dave R. Socioeconomic and cultural impact of tobacco in India. J Family Med Prim Care. 2018;7(6):1173–1176. 30613493 10.4103/jfmpc.jfmpc_36_18 PMC6293949

[8] Shahjahan M, Harun MGD, Chowdhury ABMA, Ahmed K, Khan HTA. Factors influencing the initiation of smokeless tobacco consumption among low socioeconomic community in Bangladesh: a qualitative investigation. Int Q Community Health Educ. 2017;37(3-4):181–187. 28994647 10.1177/0272684X17736244

[9] Djordjevic MV, Doran KA. Nicotine content and delivery across tobacco products. In: Henningfield JE, London ED, Pogun S, eds. Nicotine Psychopharmacology. Handbook of Experimental Pharmacology, Vol 192. Berlin, Heidelberg: Springer; 2009 . 10.1007/978-3-540-69248-5_3 19184646

[10] Kozlowski LT. Origins in the USA in the 1980s of the warning that smokeless tobacco is not a safe alternative to cigarettes: a historical, documents-based assessment with implications for comparative warnings on less harmful tobacco/nicotine products. Harm Reduct J. 2018;15(1):21. 29661189 10.1186/s12954-018-0228-8 PMC5902931

[11] Rogozinski J, Rogoziński J. Smokeless Tobacco in the Western World, 1550–1950. Westport, CT: Greenwood Publishing Group ; 1990.

[12] Sinha DN, Rizwan SA, Aryal KK, Karki KB, Zaman MM, Gupta PC. Trends of smokeless tobacco use among adults (aged 15-49 years) in Bangladesh, India and Nepal. Asian Pac J Cancer Prev. 2015;16(15):6561–6568. 26434875 10.7314/apjcp.2015.16.15.6561

[13] Siddiqi K, Husain S, Vidyasagaran A, Readshaw A, Mishu MP, Sheikh A. Global burden of disease due to smokeless tobacco consumption in adults: an updated analysis of data from 127 countries. BMC Med. 2020;18(1):222. 32782007 10.1186/s12916-020-01677-9 PMC7422596

[14] Siddiqi K, Shah S, Abbas SM, et al. Global burden of disease due to smokeless tobacco consumption in adults: analysis of data from 113 countries. BMC Med. 2015;13:194. 26278072 10.1186/s12916-015-0424-2 PMC4538761

[15] Saqib MAN, Rafique I, Qureshi H, et al. Burden of tobacco in Pakistan: findings from global adult tobacco survey 2014. Nicotine Tob Res. 2019;21(1):136. 29452373 10.1093/ntr/ntx282

[16] Sufia S, Khan AA, Ijaz S. Patterns of tobacco use in Pakistan. Pak Oral Dental J. 2003;23(1):45–50.

[17] Monika S, Dineshkumar T, Priyadharini S, Niveditha T, Sk P, Rajkumar K. Smokeless tobacco products (STPs) harbour bacterial populations with potential for oral carcinogenicity. Asian Pac J Cancer Prev. 2020;21(3):815–824. 32212812 10.31557/APJCP.2020.21.3.815 PMC7437332

[18] Niaz K, Maqbool F, Khan F, Bahadar H, Ismail Hassan F, Abdollahi M. Smokeless tobacco (paan and gutkha) consumption, prevalence, and contribution to oral cancer. Epidemiol Health. 2017;39:e2017009. 28292008 10.4178/epih.e2017009 PMC5543298

[19] Mistry R, Jones AD, Pednekar MS, et al. Antenatal tobacco use and iron deficiency anemia: integrating tobacco control into antenatal care in urban India. Reprod Health. 2018;15(1):72. 29720206 10.1186/s12978-018-0516-5 PMC5932801

[20] Pratinidhi A, Gandham S, Shrotri A, Patil A, Pardeshi S. Use of ‘Mishri’: a smokeless form of tobacco during pregnancy and its perinatal outcome. Indian J Community Med. 2010;35(1):14–18. 20606913 10.4103/0970-0218.62547 PMC2888344

[21] England LJ, Kim SY, Tomar SL, et al. Non-cigarette tobacco use among women and adverse pregnancy outcomes. Acta Obstet Gynecol Scand. 2010;89(4):454–464. 20225987 10.3109/00016341003605719 PMC5881107

[22] Inamdar AS, Croucher RE, Chokhandre MK, Mashyakhy MH, Marinho VC. Maternal smokeless tobacco use in pregnancy and adverse health outcomes in newborns: a systematic review. Nicotine Tob Res. 2015;17(9):1058–1066. 25534929 10.1093/ntr/ntu255

[23] Gupta PC, Subramoney S. Smokeless tobacco use and risk of stillbirth: a cohort study in Mumbai, India. Epidemiology. 2006;17(1):47–51. 16357594 10.1097/01.ede.0000190545.19168.c4

[24] Ashfaq M, Channa MA, Malik MA, Khan D. Morphological changes in human placenta of wet snuff users. J Ayub Med Coll Abbottabad. 2008;20(2):110–113. 19385472

[25] Gupta PC, Subramoney S, Sreevidya S. Smokeless tobacco use, birth weight, and gestational age: population based, prospective cohort study of 1217 women in Mumbai, India. BMJ. 2004;328(7455):1538. 15198947 10.1136/bmj.38113.687882.EB PMC437147

[26] Mittal S. Smoking and tobacco use: ill effects on Reproductive, Maternal, Newborn, Child Health, and Adolescent (RMNCHA) Program—a review. Ann Natl Acad Med Sci. 2019;55(02):065–073.

[27] England LJ, Levine RJ, Mills JL, Klebanoff MA, Yu KF, Cnattingius S. Adverse pregnancy outcomes in snuff users. Am J Obstet Gynecol. 2003;189(4):939–943. 14586330 10.1067/s0002-9378(03)00661-6

[28] Suliankatchi RA, Sinha DN. The human cost of tobacco chewing among pregnant women in India: a systematic review and meta-analysis. J Obstet Gynaecol India. 2016;66(suppl 1):161–166. 27651596 10.1007/s13224-015-0821-7 PMC5016430

[29] Hambidge KM, Krebs NF, Westcott JE, et al.; Preconception Trial Group. Preconception maternal nutrition: a multi-site randomized controlled trial. BMC Pregnancy Childbirth. 2014;14:111. 24650219 10.1186/1471-2393-14-111 PMC4000057

[30] Hambidge KM, Westcott JE, Garcés A, et al.; Women First Preconception Trial Study Group. A multicountry randomized controlled trial of comprehensive maternal nutrition supplementation initiated before conception: the Women First trial. Am J Clin Nutr. 2019;109(2):457–469. 30721941 10.1093/ajcn/nqy228 PMC6367966

[31] Pasha O, Saleem S, Ali S, et al. Maternal and newborn outcomes in Pakistan compared to other low and middle income countries in the Global Network’s Maternal Newborn Health Registry: an active, community-based, pregnancy surveillance mechanism. Reprod Health. 2015;12(suppl 2):S15. 26062610 10.1186/1742-4755-12-S2-S15 PMC4464035

[32] Aziz Ali S, Abbasi Z, Feroz A, et al. Factors associated with anemia among women of the reproductive age group in Thatta district: study protocol. Reprod Health. 2019;16(1):34. 30885226 10.1186/s12978-019-0688-7 PMC6423857

[33] Parks S, Hoffman MK, Goudar SS, et al. Maternal anaemia and maternal, fetal, and neonatal outcomes in a prospective cohort study in India and Pakistan. BJOG. 2019;126(6):737–743. 30554474 10.1111/1471-0528.15585 PMC6459713

[34] NoorAli R, Luby S, Rahbar MH. Does use of a government service depend on distance from the health facility? Health Policy Plan. 1999;14(2):191–197. 10538722 10.1093/heapol/14.2.191

[35] World Health Organization. Haemoglobin Concentrations for the Diagnosis of Anaemia and Assessment of Severity. 2011.

[36] Howson C, Kinney M, Lawn J. March of Dimes, PMNCH, save the children, WHO. Born too Soon: the Global Action Report on Preterm Birth. Geneva: World Health Organization; 2012.

[37] World Health Organization. Neonatal and Perinatal Mortality: Country, Regional and Global Estimates. 2006.

[38] McClure EM, Saleem S, Goudar SS, et al. Stillbirth rates in low-middle income countries 2010–2013: a population-based, multi-country study from the global network. Reprod Health. 2015;12(suppl 2):S7. 10.1186/1742-4755-12-S2-S7 PMC4464024 26063292

[39] Steyn K, de Wet T, Saloojee Y, Nel H, Yach D. The influence of maternal cigarette smoking, snuff use and passive smoking on pregnancy outcomes: the birth to ten study. Paediatr Perinat Epidemiol. 2006;20(2):90–99. 16466427 10.1111/j.1365-3016.2006.00707.x

[40] England LJ, Kim SY, Shapiro-Mendoza CK, et al. Maternal smokeless tobacco use in Alaska Native women and singleton infant birth size. Acta Obstet Gynecol Scand. 2012;91(1):93–103. 21902677 10.1111/j.1600-0412.2011.01273.x

[41] Verma RC, Chansoriya M, Kaul KK. Effect of tobacco chewing by mothers on fetal outcome. Indian Pediatr. 1983;20(2):105–111. 6862608

[42] Subramoney S, Gupta PC. Anemia in pregnant women who use smokeless tobacco. Nicotine Tob Res. 2008;10(5):917–920. 18569767 10.1080/14622200802027206

[43] McDonald CM, McLean J, Kroeun H, Talukder A, Lynd LD, Green TJ. Household food insecurity and dietary diversity as correlates of maternal and child undernutrition in rural Cambodia. Eur J Clin Nutr. 2015;69(2):242–246. 25117993 10.1038/ejcn.2014.161

[44] Ghose B, Tang S, Yaya S, Feng Z. Association between food insecurity and anemia among women of reproductive age. PeerJ. 2016;4:e1945. 27168968 10.7717/peerj.1945 PMC4860303

[45] Olson CM. Food insecurity and maternal health during pregnancy. J Am Diet Assoc. 2010;110(5):690–691. 20430129 10.1016/j.jada.2010.02.001

[46] Zhou D, Shah T, Ali S, Ahmad W, Din IU, Ilyas A. Factors affecting household food security in rural northern hinterland of Pakistan. J. Saudi Soc. 2019;18(2):201–210.

[47] Lander RL, Hambidge KM, Westcott JE, et al. Pregnant women in four low-middle income countries have a high prevalence of inadequate dietary intakes that are improved by dietary diversity. Nutrients. 2019;11(7):1560. 10.3390/nu11071560 PMC6682861 31295916

[48] Stanfill SB, Connolly GN, Zhang L, et al. Global surveillance of oral tobacco products: total nicotine, unionised nicotine and tobacco-specific N-nitrosamines. Tob Control. 2011;20(3):e2. 10.1136/tc.2010.037465 21109685

[49] World Bank. Country and Lending Groups. Washington, DC: World Bank; 2016.

[50] England LJ, Bunnell RE, Pechacek TF, Tong VT, McAfee TA. Nicotine and the developing human: a neglected element in the electronic cigarette debate. Am J Prev Med. 2015;49(2):286–293. 25794473 10.1016/j.amepre.2015.01.015 PMC4594223

